# Analysis of risk allele frequencies of single nucleotide polymorphisms related to open-angle glaucoma in different ethnic groups

**DOI:** 10.1186/s12920-021-00921-2

**Published:** 2021-03-16

**Authors:** Hyun-Tae Shin, Byung Woo Yoon, Je Hyun Seo

**Affiliations:** 1 Veterans Health Service Medical Center, Veterans Medical Research Institute, Jinhwangdo-ro 61-gil 53,Gangdong-gu, Seoul, 05368 Korea; 2 Department of Dermatology, Inha University School of Medicine, Inha-ro 100, Michuhol-gu, Incheon, 22212 Korea; 3 Division of Oncology, Department of Internal Medicine, Inje University Seoul Paik Hospital, Mareunnae-ro 9,Jung-gu, Seoul, 04551 Korea

**Keywords:** Open-angle glaucoma, Allele frequency, Single nucleotide polymorphism, Intraocular pressure, Prevalence, Normal-tension glaucoma

## Abstract

**Background:**

The prevalence of open-angle glaucoma (OAG) varies from 0.5% to 7.0% among populations of diverse ancestry, suggesting the existence of genetic differences. The purposes of this study were to provide insights into genetic causes of OAG, which can result in prevalence and phenotype differences among populations of diverse ancestry for OAG, and to compare allele frequencies of intraocular pressure (IOP) elevation-related SNPs in OAG among Koreans and other ethnic groups.

**Methods:**

We collected the data on a total of 135 OAG-associated single nucleotide polymorphisms (SNPs) from a genome-wide association studies (GWAS) catalog. The population-level allele frequencies of these SNPs were derived based on the 1000 Genomes Project and Korean Reference Genome Database. We used Fisher's exact test to assess whether the effect allele at a given SNP was significantly enriched or depleted.

**Results:**

European, American, and South Asian populations showed similar heatmap patterns, while African, East Asian, and Korean populations had distinct patterns. Korean population presented different profiles compared to other groups; rs1579050 (*FMNL2* gene), rs2024211 (*CAV2;CAV1*), and rs8141433 (*GNB1L*;*TXNRD2* gene), which are known to be associated with IOP variation, were enriched in Americans, Europeans, and Africans, and depleted in Koreans. These can be the candidates for the causative genes of differences in the prevalence of IOP variation in OAG according to ethnic groups.

**Conclusions:**

Differences in allele frequencies associated with IOP related SNPs between Koreans and other ethnicities were observed, which may explain the high prevalence of OAG with normal IOP predominantly in Koreans and East Asians.

**Supplementary information:**

The online version contains supplementary material available at 10.1186/s12920-021-00921-2.

## Background

Glaucoma, which is a progressive optic neuropathy characterized by degeneration of the retinal ganglion cells and their axons and a corresponding visual field defect [1], is a leading cause of irreversible blindness worldwide. Prevalence of glaucoma varies among ethnicity and regions, from 0.5% to 8.0% for open-angle glaucoma (OAG) [2–5], and from 0.1% to 2.3% for angle-closure glaucoma [6–8], suggesting the existence of genetic differences among populations of diverse ancestry. As OAG is the predominant form of glaucoma, epidemiologic research regarding diverse ancestry populations has been conducted [2, 5, 9–12]. According to a meta-analysis report [13], the prevalence of OAG was higher in Africans, followed by Europeans and Asians. Still, there were little data on other ethnic groups, such as South and East Asians. A recent study complementing this has shown that the prevalence of OAG was higher in Africans and Latinos, followed by Europeans, South Asians, and East Asians [14]. The relationship between the prevalence and geographical variations might encompass genetic, environmental, and cultural differences.

Evidence for a causal relationship between elevated intraocular pressure (IOP) and OAG is robust, but normal-tension glaucoma (NTG) is considered a part of the spectrum of OAG intrinsically, which is arbitrarily based on the distribution of IOP; OAG with IOP ≤ 21 mmHg is termed as NTG. Specifically, NTG is common in Asians, including Koreans (77.0%) and comprises the majority (52–92%) of cases of OAG in Asian studies [15–18]; the proportions are higher than those in Europeans (30–38%) and African ancestry populations (57.1%) [3, 19, 20]. This difference in the pattern of prevalence of NTG according to populations of diverse ancestry suggested that it may be related to different allele frequencies of glaucoma-related single nucleotide polymorphism (SNP). In addition, first-degree relatives of affected patients are estimated to have as much as 4 to 10 times increased risk compared to the general population [21, 22]. These suggest that genetic aspects should be considered as a cause of glaucoma. Recently, studies based on genome-wide association study (GWAS) of glaucoma with meta-analysis using the results from various cohorts such as International Glaucoma Genetics Consortium (IGGC), Australian and New Zealand Registry of Advanced Glaucoma (ANZRAG), and UK Biobank (UKBB) had revealed novel risk loci and SNPs related with IOP [23–26]. Combining these results using the GWAS catalog (NHGRI-EBI) [27] and utilizing 1000 Genomes Project phase 3 data [28], we could infer the difference in glaucoma-related SNPs according to populations of diverse ancestry. Additionally, it is possible to assess the allele frequency of OAG-associated SNPs and IOP elevation-associated SNPs in OAG among Koreans using 1722 whole-genome sequencing data of healthy Koreans in the Korean Reference Genome Database (KRGDB) [29].

Hence, the purposes of this study were to gain insights into genetic causes of OAG, which can result in prevalence and phenotype differences among populations of diverse ancestry for OAG, and to compare allele frequencies of IOP elevation-related SNPs in OAG among Koreans and other population groups. We also developed the composite genetic risk score for OAG as a whole and OAG with high IOP and tested the correlation between a population-level average of composite risk scores and OAG prevalence.

## Methods

This study was approved and monitored by the Institutional Review Board (IRB) of the Veterans Health Service Medical Center, Korea (IRB No. 2019-07-008).

### Comparison of OAG-related SNPs in global population and East Asians

According to the International Society of Geographical and Epidemiological Ophthalmology (ISGEO) classification [30], OAG is defined as glaucomatous optic neuropathy in the presence of an open angle and no other ocular abnormality accountable for the neuropathy. We researched the GWAS catalog (NHGRI-EBI, https://www.ebi.ac.uk/gwas/docs/file-downloads, “All associations v1.0.2—with added ontology annotations, GWAS Catalog study accession numbers and genotyping technology”, December 2019) for SNPs that were associated with ‘open-angle glaucoma’-related trait (EFO_0004190). Initially, 146 glaucoma-associated SNPs from the GWAS catalog were collected. Of these, a total of 135 SNPs was used for analysis after removing the repeated ones (Additional files 2, 3: Table S1 and Table S2).

Among SNPs associated with OAG-related trait, we determined OAG risk by examining the sign of beta-coefficient, whether the odds ratio for the effect allele was greater than one, and text description in the primary GWAS reports. The details and advantages of the method have been described elsewhere [31]. In brief, the population-level allele frequencies of these SNPs were derived based on the 1000 Genomes Project phase 3 (n = 2504) and KRGDB (n = 1722). The 1000 Genomes Project surveys genetic variations among 2504 individuals from 26 worldwide populations, which can be grouped into African (AFR), East Asian (EAS), European (EUR), South Asian (SAS), and the American (AMR) based on their geographical locations and ancestries [28]; the data were downloaded from ftp://ftp.1000genomes.ebi.ac.uk/vol1/ftp/release/20130502/ (last accessed: January 15, 2020). Because the East Asian data in the 1000 Genomes Project did not include data from the Korean population, we compared the data from the five continents with data extracted from KRGDB, which included the whole genome sequencing data for 1722 Korean (KOR) individuals [29]. The data on the population frequency of the SNPs were downloaded from the web-based database (http://152.99.75.168:9090/KRGDB/menuPages/download.jsp/, last accessed: January 15, 2020). For the comparison of the distribution of individual risk alleles of the Korean population, individual genotype results of the 2nd phase of KRGDB (n = 1099) were obtained from the National Human Resource Bank of Korea.

### Comparison of SNPs related to IOP elevation in OAG in Global population and East Asians

OAG is defined as an optic nerve state, but IOP is considered a major risk factor. Hence, research for allele frequencies of SNPs influencing IOP may give us insights on the difference in the prevalence of NTG and OAG with high IOP according to different ancestries. The 52 SNPs related to IOP are shown in Table 1, which have been obtained from GWAS catalog data, a study using Genetic Epidemiology Research in Adult Health and Aging (GERA) cohort [32], a large multi-ethnic study for identifying novel loci related to IOP [33], and meta-analysis results of IGGC, ANZRAG, and UKBB [23–26]. The population-level allele frequencies of these SNPs were derived as described above.

**Table 1:** Effect allele frequencies (EAFs) of intraocular pressure related single nucleotide polymorphisms in populations of diverse ancestry including Koreans

SNP ID	Chr	Position	Type	Ref Allele	Alt Allele	Nearby/containing Gene	Global EAF	AMR EAF	AMR log_10_ *P*	AFR EAF	AFR log_10_ *P*	EAS EAF
rs1013278	chr7	117603820	Intergenic	G	C	CTTNBP2;LSM8	0.31	0.28	− 0.735	0.33	0.738	0.1
rs10281637	chr7	116151338	Intergenic	T	C	CAV2;CAV1	0.22	0.21	− 0.177	0.4	36.671	0.0089
rs10483727	chr14	61072875	Intergenic	T	C	SIX6;SALRNA1	0.35	0.66	51.990	0.034	− 146.666	0.21
rs10505100	chr8	108278616	Intronic	C	A	ANGPT1	0.16	0.18	0.569	0.11	− 5.368	0.23
rs10918274	chr1	165714416	Intronic	T	C	TMCO1	0.92	0.87	− 3.857	0.92	0.000	0.99
rs11217878	chr11	120340383	Intronic	G	A	ARHGEF12	0.21	0.15	− 3.286	0.25	2.601	0.24
rs113985657	chr6	597203	Intronic	C	T	EXOC2	0.12	0.11	− 0.249	0.087	− 3.132	0.14
rs11710139	chr3	15005942	Intergenic	G	A	LINC01214;TSC22D2	0.16	0.14	− 0.578	0.16	0.010	0.075
rs12377624	chr9	129373110	Intergenic	G	C	MVB12B;LMX1B	0.23	0.24	0.194	0.11	− 23.178	0.13
rs1254276	chr14	60847001	Intergenic	C	T	LINC02322;C14orf39	0.63	0.32	− 51.921	0.89	82.782	0.78
rs12699251	chr7	11679113	Intronic	A	G	THSD7A	0.25	0.35	6.518	0.11	− 29.965	0.2
rs1579050	chr2	153364527	Intronic	A	G	FMNL2	0.28	0.46	19.037	0.1	− 46.833	0.026
rs17752199	chr6	51406848	Intergenic	A	G	TFAP2B;PKHD1	0.13	0.13	0.000	0.15	1.166	0.12
rs1874458	chr16	65080739	Intronic	G	A	CDH11	0.2	0.21	0.187	0.085	− 24.499	0.21
rs2022945	chr8	108251139	Intergenic	A	G	ABRA;ANGPT1	0.84	0.83	− 0.204	0.9	7.594	0.77
rs2024211	chr7	116153025	Intergenic	A	C	CAV2;CAV1	0.21	0.2	− 0.177	0.34	20.570	0.0089
rs2073006	chr6	637465	Intronic	C	T	EXOC2	0.11	0.12	0.258	0.045	− 13.535	0.15
rs2188836	chr7	117635382	Intergenic	C	T	CTTNBP2;LSM8	0.33	0.28	− 1.730	0.39	4.233	0.09
rs2317961	chr6	1533116	Intergenic	A	G	FOXF2;FOXCUT	0.63	0.59	− 1.121	0.82	40.735	0.51
rs2472493	chr9	107695848	Intergenic	G	A	ABCA1;SLC44A1	0.61	0.65	1.095	0.69	6.970	0.49
rs2472496	chr9	107695353	Intergenic	G	A	ABCA1;SLC44A1	0.61	0.64	0.690	0.69	6.970	0.47
rs2487032	chr9	107703934	Intergenic	G	A	ABCA1;SLC44A1	0.6	0.48	− 7.836	0.83	58.058	0.49
rs2745572	chr6	1548369	Intergenic	A	G	FOXF2;FOXCUT	0.36	0.38	0.395	0.15	− 52.096	0.49
rs28500712	chr4	7896213	Intronic	A	G	AFAP1	0.66	0.59	− 3.016	0.72	4.381	0.68
rs28520091	chr4	7846240	Intronic	C	T	AFAP1	0.32	0.38	2.332	0.15	− 36.294	0.29
rs28795989	chr4	7891545	Intronic	A	G	AFAP1	0.29	0.32	0.743	0.14	− 30.033	0.18
rs2935057	chr6	170454915	Intergenic	A	G	LINC00574;LOC102724511	0.8	0.82	0.502	0.74	− 5.373	0.76
rs3013274	chr6	170464367	Intergenic	G	A	LINC00574;LOC102724511	0.61	0.58	− 0.664	0.7	8.654	0.5
rs31918	chr5	14820927	Intronic	C	T	ANKH	0.29	0.34	1.752	0.28	− 0.298	0.33
rs327716	chr7	80838977	Intergenic	A	G	SEMA3C;LOC105369146	0.71	0.68	− 0.743	0.81	12.896	0.91
rs33912345	chr14	60976537	Exonic	C	A	SIX6	0.34	0.66	54.967	0.032	− 143.370	0.21
rs3785176	chr16	8896931	Intronic	A	C	PMM2	0.18	0.14	− 1.712	0.096	− 13.535	0.28
rs4141671	chr10	60338753	Intronic	T	C	BICC1	0.49	0.32	− 15.716	0.55	3.871	0.48
rs4236601	chr7	116162729	Intergenic	G	A	CAV2;CAV1	0.23	0.21	− 0.434	0.4	32.449	0.0099
rs55892100	chr7	115810676	Intergenic	A	G	TFEC;TES	0.54	0.43	− 6.518	0.61	5.149	0.8
rs5756813	chr22	38175477	Intergenic	G	T	TRIOBP;H1F0	0.55	0.62	2.799	0.4	− 21.169	0.71
rs58073046	chr11	120248493	Intronic	A	G	ARHGEF12	0.1	0.042	− 6.316	0.013	− 32.104	0.17
rs61394862	chr5	14851094	Intronic	C	T	ANKH	0.28	0.33	1.781	0.26	− 0.777	0.32
rs6478746	chr9	129367398	Intergenic	G	A	MVB12B;LMX1B	0.8	0.85	2.421	0.73	− 6.990	0.97
rs66602224	chr8	108293718	Intronic	G	A	ANGPT1	0.32	0.32	0.000	0.44	14.831	0.14
rs6732795	chr2	69411517	Intronic	A	C	ANTXR1	0.66	0.49	− 16.002	0.78	16.515	0.85
rs73174345	chr3	169252883	Intronic	T	G	MECOM	0.054	0.036	− 1.095	0.13	18.287	0
rs746491	chr11	86406159	Intergenic	C	A	ME3;PRSS23	0.15	0.17	0.588	0.11	− 3.715	0.14
rs7518099	chr1	165736880	Intronic	C	T	TMCO1	0.92	0.87	− 3.857	0.91	− 0.608	0.99
rs7555523	chr1	165718979	Intronic	C	A	TMCO1	0.9	0.87	− 1.399	0.85	− 6.009	0.99
rs7924522	chr11	128380742	Intronic	C	A	ETS1	0.75	0.71	− 1.291	0.79	2.503	0.86
rs8141433	chr22	19854006	Intergenic	A	G	GNB1L;TXNRD2	0.28	0.16	− 10.572	0.69	159.128	0.037
rs9284802	chr3	85095766	Intronic	G	A	CADM2	0.32	0.33	0.177	0.13	− 46.150	0.17
rs945686	chr9	129378026	Intronic	G	C	LMX1B	0.82	0.87	2.585	0.73	− 11.619	0.99
rs9494457	chr6	136474794	Intronic	T	A	PDE7B	0.39	0.45	2.158	0.37	− 0.696	0.33
rs9853115	chr3	186131600	Intergenic	T	A	DGKG;LINC02052	0.56	0.47	− 4.386	0.48	− 6.471	0.73
rs9913911	chr17	10031183	Intronic	A	G	GAS7	0.31	0.32	0.177	0.16	− 28.132	0.5

SNP ID	Chr	Position	Type	Ref Allele	Alt Allele	Nearby/containing Gene	EAS log_10_ *P*	SAS EAF	SAS log_10_ *P*	EUR EAF	EUR log_10_ *P*	KOR EAF	KOR log_10_ *P*
rs1013278	chr7	117603820	Intergenic	G	C	CTTNBP2;LSM8	− 47.563	0.4	6.676	0.4	7.043	0.114	− 102.924
rs10281637	chr7	116151338	Intergenic	T	C	CAV2;CAV1	− 80.906	0.19	− 1.271	0.26	2.100	0.007	− 232.094
rs10483727	chr14	61072875	Intergenic	T	C	SIX6;SALRNA1	− 17.809	0.42	4.030	0.6	47.063	0.271	− 13.494
rs10505100	chr8	108278616	Intronic	C	A	ANGPT1	6.644	0.2	2.350	0.11	− 4.159	0.283	40.539
rs10918274	chr1	165714416	Intronic	T	C	TMCO1	20.362	0.96	5.017	0.86	− 7.783	0.993	62.418
rs11217878	chr11	120340383	Intronic	G	A	ARHGEF12	1.395	0.23	0.659	0.16	− 3.413	0.227	1.147
rs113985657	chr6	597203	Intronic	C	T	EXOC2	1.036	0.12	0.000	0.14	1.014	0.208	26.399
rs11710139	chr3	150059342	Intergenic	G	A	LINC01214;TSC22D2	− 12.606	0.16	0.000	0.23	6.403	0.096	− 16.719
rs12377624	chr9	129373110	Intergenic	G	C	MVB12B;LMX1B	− 12.549	0.33	9.258	0.37	18.120	0.079	− 78.578
rs1254276	chr14	60847001	Intergenic	C	T	LINC02322;C14orf39	19.784	0.6	− 0.943	0.4	− 39.746	0.727	19.784
rs12699251	chr7	11679113	Intronic	A	G	THSD7A	− 3.064	0.25	0.000	0.4	19.691	0.229	− 1.524
rs1579050	chr2	153364527	Intronic	A	G	FMNL2	− 89.083	0.35	4.372	0.58	69.618	0.025	− 242.856
rs17752199	chr6	51406848	Intergenic	A	G	TFAP2B;PKHD1	− 0.364	0.12	− 0.327	0.12	− 0.320	0.103	− 3.773
rs1874458	chr16	65080739	Intronic	G	A	CDH11	0.314	0.2	0.000	0.33	17.034	0.250	7.076
rs2022945	chr8	108251139	Intergenic	A	G	ABRA;ANGPT1	− 6.644	0.8	− 2.350	0.89	4.159	0.723	− 37.209
rs2024211	chr7	116153025	Intergenic	A	C	CAV2;CAV1	− 76.273	0.19	− 0.664	0.26	3.118	0.007	− 218.926
rs2073006	chr6	637465	Intronic	C	T	EXOC2	3.236	0.11	0.000	0.13	1.060	0.220	40.976
rs2188836	chr7	117635382	Intergenic	C	T	CTTNBP2;LSM8	− 60.936	0.43	7.936	0.41	5.477	0.095	− 150.366
rs2317961	chr6	1533116	Intergenic	A	G	FOXF2;FOXCUT	− 11.567	0.52	− 8.961	0.64	0.217	0.475	− 44.116
rs2472493	chr9	107695848	Intergenic	G	A	ABCA1;SLC44A1	− 11.416	0.65	1.567	0.58	− 1.060	0.460	− 40.945
rs2472496	chr9	107695353	Intergenic	G	A	ABCA1;SLC44A1	− 15.152	0.67	3.051	0.58	− 1.060	0.428	− 59.932
rs2487032	chr9	107703934	Intergenic	G	A	ABCA1;SLC44A1	− 9.692	0.56	− 1.474	0.5	− 7.924	0.476	− 28.298
rs2745572	chr6	1548369	Intergenic	A	G	FOXF2;FOXCUT	13.501	0.48	10.620	0.36	0.000	0.517	45.723
rs28500712	chr4	7896213	Intronic	A	G	AFAP1	0.586	0.54	− 10.735	0.72	3.416	0.709	5.547
rs28520091	chr4	7846240	Intronic	C	T	AFAP1	− 1.186	0.37	2.314	0.5	25.369	0.295	− 1.770
rs28795989	chr4	7891545	Intronic	A	G	AFAP1	− 12.655	0.26	− 1.084	0.6	73.748	0.170	− 36.886
rs2935057	chr6	170454915	Intergenic	A	G	LINC00574;LOC102724511	− 2.238	0.84	2.252	0.88	8.761	0.729	− 13.364
rs3013274	chr6	170464367	Intergenic	G	A	LINC00574;LOC102724511	− 9.753	0.68	4.063	0.57	− 1.606	0.428	− 59.784
rs31918	chr5	14820927	Intronic	C	T	ANKH	1.881	0.22	− 4.799	0.29	0.000	0.334	4.776
rs327716	chr7	80838977	Intergenic	A	G	SEMA3C;LOC105369146	45.585	0.64	− 4.367	0.43	− 60.462	0.947	183.742
rs33912345	chr14	60976537	Exonic	C	A	SIX6	− 15.632	0.4	3.083	0.6	50.841	0.268	− 11.500
rs3785176	chr16	8896931	Intronic	A	C	PMM2	11.463	0.13	− 3.575	0.25	6.099	0.316	45.623
rs4141671	chr10	60338753	Intronic	T	C	BICC1	− 0.226	0.56	3.867	0.49	0.000	0.489	− 0.037
rs4236601	chr7	116162729	Intergenic	G	A	CAV2;CAV1	− 84.231	0.19	− 1.952	0.26	1.262	0.007	− 244.996
rs55892100	chr7	115810676	Intergenic	A	G	TFEC;TES	54.802	0.43	− 8.650	0.37	− 21.708	0.835	182.195
rs5756813	chr22	38175477	Intergenic	G	T	TRIOBP;H1F0	20.456	0.5	− 2.095	0.58	0.989	0.643	16.731
rs58073046	chr11	120248493	Intronic	A	G	ARHGEF12	8.745	0.21	17.513	0.1	0.000	0.168	18.725
rs61394862	chr5	14851094	Intronic	C	T	ANKH	1.910	0.21	− 4.971	0.28	0.000	0.333	6.636
rs6478746	chr9	129367398	Intergenic	G	A	MVB12B;LMX1B	50.342	0.78	− 0.668	0.7	− 10.654	0.967	127.390
rs66602224	chr8	108293718	Intronic	G	A	ANGPT1	− 32.704	0.28	− 1.639	0.4	5.548	0.204	− 31.901
rs6732795	chr2	69411517	Intronic	A	C	ANTXR1	34.845	0.68	0.540	0.4	− 50.841	0.900	152.394
rs73174345	chr3	169252883	Intronic	T	G	MECOM	− 21.410	0.014	− 7.882	0.059	0.230	0.000	− 59.854
rs746491	chr11	86406159	Intergenic	C	A	ME3;PRSS23	− 0.338	0.13	− 0.815	0.21	5.097	0.193	6.653
rs7518099	chr1	165736880	Intronic	C	T	TMCO1	20.362	0.96	5.017	0.86	− 7.783	0.993	63.362
rs7555523	chr1	165718979	Intronic	C	A	TMCO1	27.970	0.96	9.258	0.86	− 3.391	0.993	85.262
rs7924522	chr11	128380742	Intronic	C	A	ETS1	14.150	0.75	0.000	0.63	− 13.128	0.907	78.316
rs8141433	chr22	19854006	Intergenic	A	G	GNB1L;TXNRD2	− 78.658	0.22	− 3.655	0.15	− 18.042	0.040	− 200.793
rs9284802	chr3	85095766	Intronic	G	A	CADM2	− 22.155	0.41	6.585	0.63	72.241	0.176	− 49.830
rs945686	chr9	129378026	Intronic	G	C	LMX1B	61.221	0.79	− 1.348	0.76	− 4.495	0.987	156.676
rs9494457	chr6	136474794	Intronic	T	A	PDE7B	− 3.323	0.44	2.158	0.37	− 0.570	0.282	− 23.733
rs9853115	chr3	186131600	Intergenic	T	A	DGKG;LINC02052	23.247	0.57	0.193	0.53	− 1.014	0.701	38.881
rs9913911	chr17	10031183	Intronic	A	G	GAS7	28.572	0.27	− 1.662	0.37	3.416	0.554	109.722

Chr: chromosome EAF: effect allele frequency ref allele: reference allele alt allele: alterative allele AMR: Americans AFR: Africans, EAS: East Asians, SAS: South Asians, EUR: Europeans, KOR: Koreans, *P*-value: adjusted Fischer’s test, statistical significance was set at *P* < 0.05 and |log_10_ *P*|> 1.301

### Composite genetic risk score calculation using SNPs related to OAG and OAG with high IOP

To compare the composite genetic risk of OAG, we adopted the equation suggested by Mao et al*.* [31] The composite genetic risk score is calculated using the following formula:

$$Genetic\, risk\, score=\frac{\sum_{i=1}^{I}Xi}{2I}$$

where “I” refers to the number of OAG-related SNPs, and “Xi” refers to copies of risk alleles (Xi ∈ {0,1,2}) at the ith SNP. In one extreme case, if a person has two copies of risk alleles at each OAG-related SNP, then the person’s risk score will become 1. On the other hand, if a person has no copy of risk alleles at each OAG-related SNP, then the person’s risk score will become 0. A person with a composite score of 1 has the highest possible genetic risk for OAG, while a person with a score of 0 has the lowest possible genetic risk. If copies of effect alleles (0/1/2) are randomly assigned to each SNP, the expected value of the risk score will be 0.5. SNPs with a frequency difference of more than 10% between the total (n = 1722) and the 2nd phase (n = 1099) data of KRGDB were excluded from the genetic score calculation. We used the average of composite genetic risk scores for the populations for correlation with the country-wise OAG prevalence data. In addition, the composite genetic risk score was calculated using IOP elevation-related SNPs for OAG with high IOP. The prevalence of OAG with high IOP was calculated as (1—the NTG proportion of OAG [12]) × the total OAG prevalence. The correlation analysis with the composite genetic risk score and the prevalence of OAG with high IOP was performed.

### Data analyses

We used the Kruskal–Wallis rank-sum test to assess OAG related SNP frequencies according to populations of diverse ancestry and Fisher's exact test to assess whether the effect allele at a given SNP is significantly enriched or depleted compared to the global population frequency in the 1000 Genomes Project database, and the *P* values were first log_10_-transformed. In the heatmap generated to visualize allele enrichment or depletion patterns in different populations, red and purple colors meant higher and lower frequencies than the global average, respectively. If the effect allele of an SNP is enriched in a population, then the negative of log_10_ of the enrichment *P*-value (a positive number) was used to represent the SNP associated with that population in a heatmap. On the other hand, if the allele of an SNP is depleted in a population, the value of log_10_ of the depletion *P*-value (a negative number) was used to represent the SNP for that population in the heatmap. Statistical analyses were performed using R software version 3.6.0 (R Foundation, Vienna, Austria). Statistical significance was set at *P* < 0.05 and |log_10_ *P*|> 1.301.

## Results

### Patterns of OAG risk alleles among populations

**Fig. 1:**
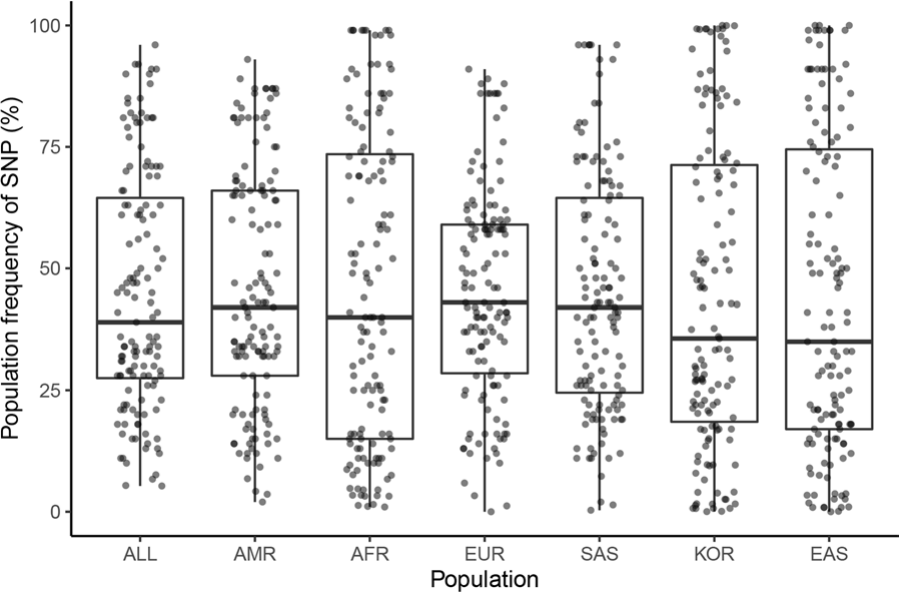
Comparison of frequency of open-angle glaucoma (OAG)-related single nucleotide polymorphisms (SNP) according to populations of diverse ancestry. This figure shows no difference in OAG-related SNP frequency between populations with the Kruskal–Wallis rank-sum test (*P* = 0.8893). AMR: American, EUR: Europeans, SAS: South Asians, AFR: Africans, EAS: East Asians, KOR: Koreans

A total of 135 OAG-related SNPs was obtained from 24 GWAS studies. Among these, 15 studies were performed in Europeans, 13 in East Asians, 4 in South Asians, 7 in Africans, and 2 in Americans (9 studies were performed in mixed ethnic populations). Clearly, populations except Europeans were understudied. However, there was no significant difference in the SNP frequency among these populations with the Kruskal–Wallis rank-sum test (Fig. 1). This result suggests that many SNPs found in Europeans are also applicable to other populations. Following collecting the OAG-associated SNPs, we obtained their effect allele frequencies (EAFs) in each of the continental groups and Koreans based on genotype information from the 1000 Genomes Project and KRGDB (Additional file 2: Table 1). A heatmap showed how significantly the effect allele was enriched or depleted across the Korean and continental groups (Additional files 1: Figure S1) with a log scale among 135 OAG-related SNPs. For Koreans, 63 OAG-related SNPs were significantly enriched, 63 SNPs were depleted, and 9 SNPs were similar to global EAF. The heatmap visualized the proportion of effect alleles in each continental group compared to the global average. There were certain patterns of risk allele frequencies depending on continental groups. The hierarchical clustering tree showed the differences among the populations; Europeans, Americans, and South Asians were in one cluster, and Africans, East Asians, and Koreans were in another cluster. In addition, we compared the EAFs of East Asians and Koreans (Additional file 3: Table S2, Additional file 1: Figure S2). Although the EAF was not much different among East Asians, 25 SNPs were enriched, 26 SNPs were depleted, and 84 SNPs were similar to those in Koreans. Moreover, the heatmap clearly showed that the main pattern of the allele frequency in East Asians in the 1000 Genomes Project was very similar to that in Koreans, while few alleles showed the opposite pattern in allele frequency between East Asians and Koreans.

### Patterns of IOP elevation-related OAG risk alleles among populations

**Fig. 2:**
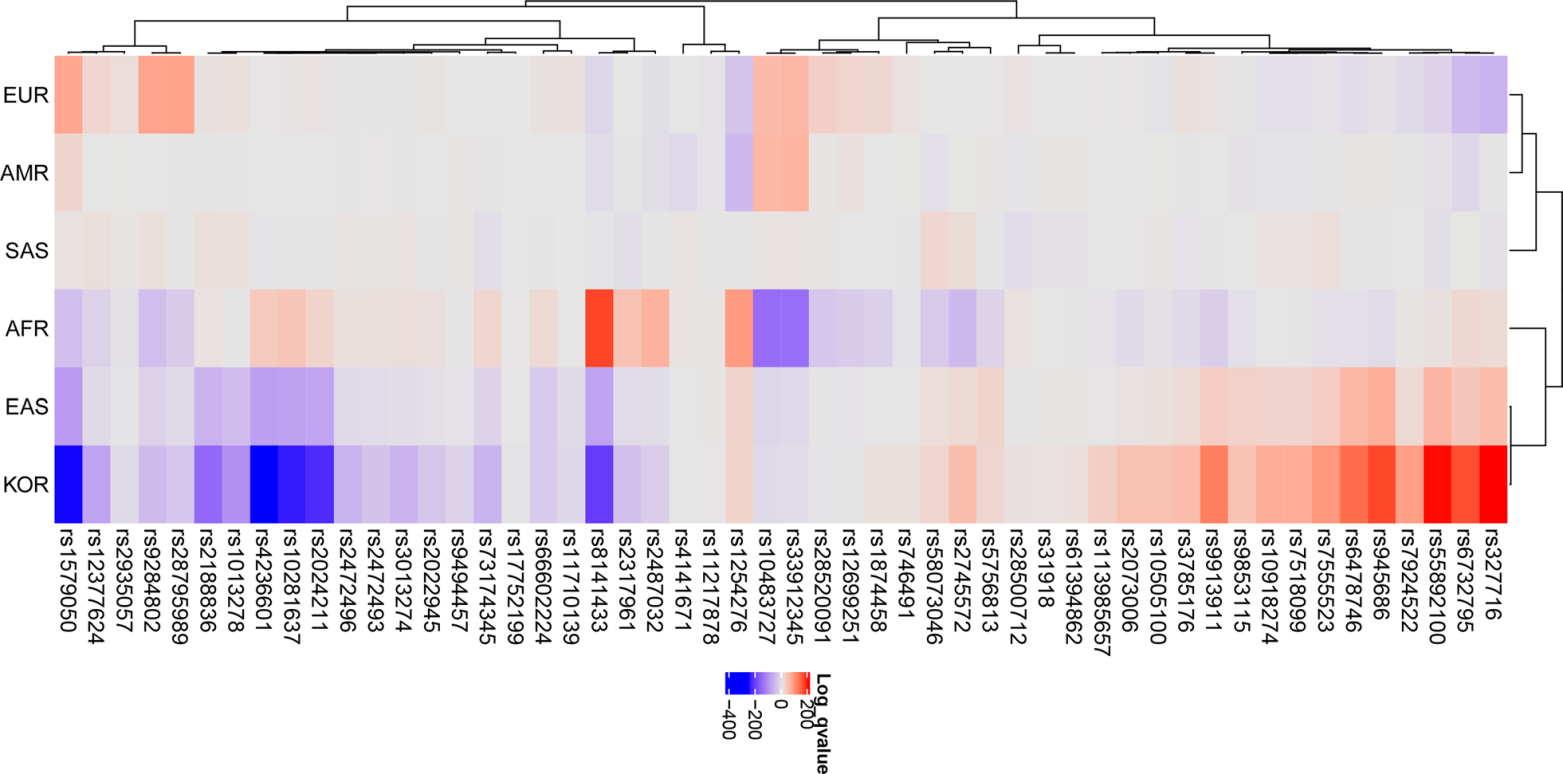
The heatmap generated using intraocular pressure elevation in open-angle glaucoma-related single nucleotide polymorphisms in the global population. The heatmap shows how significantly the effect alleles are enriched or depleted in each population. Each row shows SNPs, and each column shows populations of diverse ancestry. Red color means effect allele is enriched, whereas purple color means effect allele is depleted (log_10_ *P* > 1.301 indicated enrichment, log_10_ *P* <  − 1.301 indicated depletion). A hierarchical clustering tree shows the differences among continents; EUR, AMR, and SAS are in one cluster, and AFR, EAS, and KOR are in another cluster. AMR: American, EUR: Europeans, SAS: South Asians, AFR: Africans, EAS: East Asians, KOR: Koreans

**Fig. 3:**
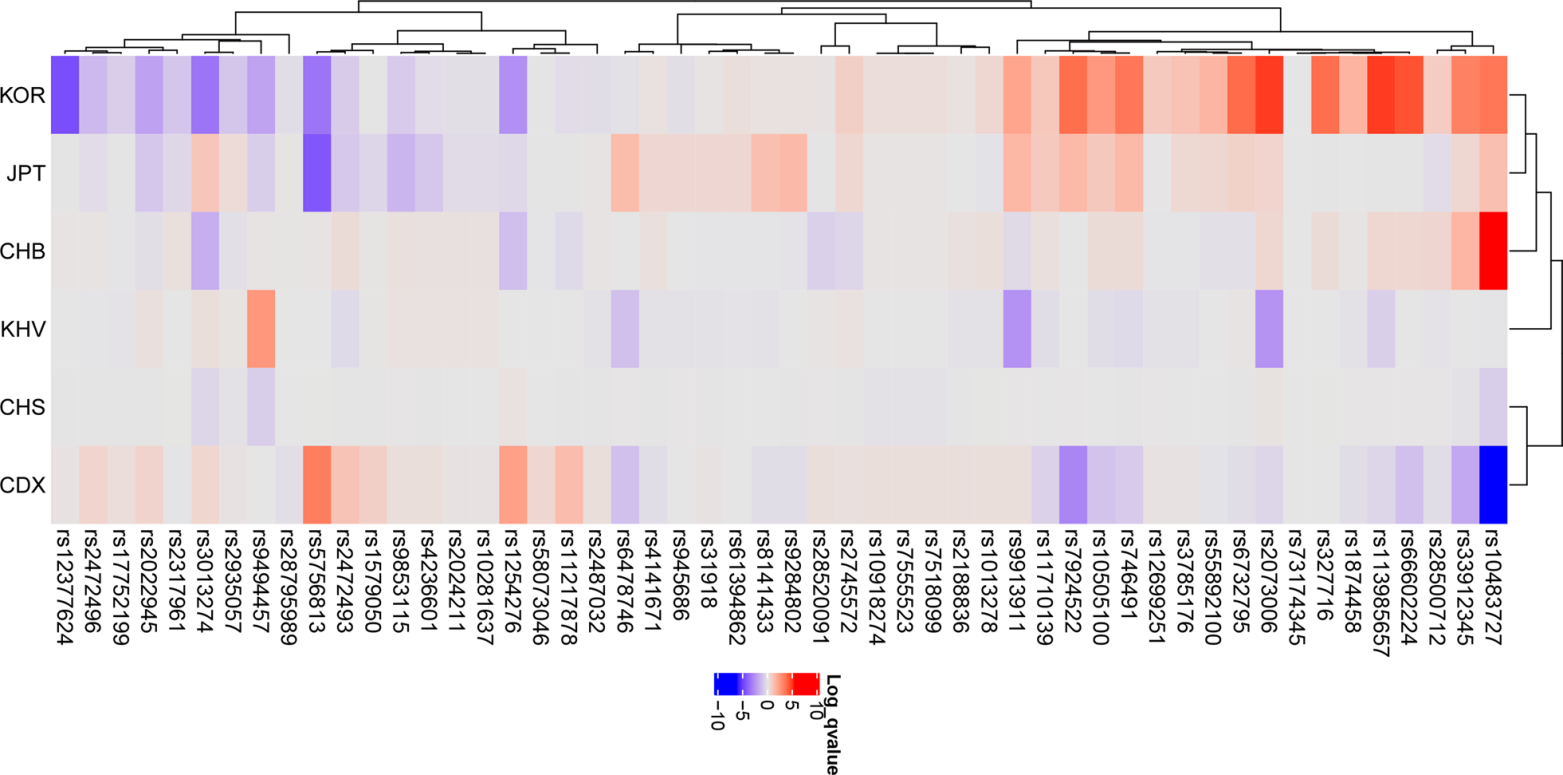
The heatmap generated using intraocular pressure elevation in open-angle glaucoma-related single nucleotide polymorphisms in the East Asian population. The heatmap shows how significantly the effect alleles are enriched or depleted in each population. Each row shows SNPs, and each column shows populations of diverse ancestry. Red color means effect allele is enriched, whereas purple color means effect allele is depleted (log_10_ *P* > 1.301 indicated enrichment, log_10_ *P* <  − 1.301 indicated depletion). A hierarchical clustering tree shows the differences among East Asians, KOR, JPT, and CHB in one cluster, and CHS, and CDX in another cluster. CDX: Chinese Dai in Xishuangbanna, CHB: Han Chinese in Beijing, China, CHS: Southern Han Chinese, China, JPT: Japanese in Tokyo, Japan, KOR: Korean in Republic Korea, KHV: Kinh in Ho Chi Minh City, Vietnam

Fifty-two IOP-related SNP traits were selected from 135 OAG-related SNPs (Table 1), and a heatmap showing how significantly the EAF was enriched or depleted across the Koreans and continental groups (Fig. 2) with a log scale among 52 IOP-related SNPs was generated. For Koreans, 23 OAG-related SNPs were significantly enriched, 27 SNPs depleted, and 2 SNPs were similar to global EAF. For example, rs2024211, located in the Intergenic of *CAV2; CAV1* that is expressed in the trabecular meshwork cells cultured from OAG eyes and encoding Caveolins [34], has T/C alleles in which the C allele was tested in European populations to identify the risk of OAG with high IOP [25]. The C allele frequencies were 26%, 36%, and 0.7% in European, African, and Korean populations, respectively. Further, rs1579050, located in *FMNL2* gene belongs to the formin-related family of proteins that acts as a downstream effector of CDC42 (Rho family member) [33], has A/G alleles in which the G allele frequencies were 58%, 10%, and 2.5% in European, African, and Korean populations. The rs8141433, located in the Intergenic of *GNB1L; TXNRD2, TXNRD2* gene encodes a mitochondrial protein required for redox homeostasis [35], revealed the G allele frequencies of 15%, 69%, and 4% in European, African, and Korean populations. A hierarchical clustering tree showed the differences among the populations; Europeans, Americans, and South Asians were in one cluster, and Africans, East Asians, and Koreans were in another cluster. These allele frequencies may be related to a difference in the NTG proportion of OAG among the populations. In addition, a heatmap showed how significantly the EAF was enriched or depleted across the Koreans and East Asians (Table 2, Fig. 3). For Koreans, rs12377624, *LMX1B* gene had G/C alleles in which the C allele frequencies were 14%, 12.5%, and 7.9% in Chinese, Japanese, and Koreans, respectively, whereas, rs2073006, *EXOC2* gene had C/T alleles in which the T allele frequencies were 11.8%, 8.1%, and 22.0% in Chinese, Vietnamese, and Koreans, respectively. However, the EAF was not much different among East Asians; for Koreans, 13 SNPs were enriched, 7 SNPs were depleted, and 32 SNPs were similar to those in Global East Asians. A hierarchical clustering tree showed the differences among East Asians, KOR, Japanese, and Han Chinese in Beijing in one cluster, and Southern Han Chinese and Chinese Dai in Xishuangbanna in another cluster.

**Table 2:** Effect allele frequencies (EAFs) of intraocular pressure related single nucleotide polymorphisms in East Asian groups including Koreans

SNP ID	Chr	Position	Type	Ref Allele	Alt Allele	Nearby/containing Gene	Global East Asian EAF	CHS EAF	CHS log_10_ *P*	CDX EAF	CDX log_10_ *P*	KHV EAF	KHV log_10_ *P*
rs1013278	chr7	117603820	Intergenic	G	C	CTTNBP2;LSM8	0.1	0.105	0.000	0.118	0.162	0.076	− 0.079
rs10281637	chr7	116151338	Intergenic	T	C	CAV2;CAV1	0.0089	0.005	0.000	0.011	0.061	0.015	0.079
rs10483727	chr14	61072875	Intergenic	T	C	SIX6;SALRNA1	0.21	0.167	− 0.381	0.113	− 5.663	0.202	− 0.034
rs10505100	chr8	108278616	Intronic	C	A	ANGPT1	0.23	0.233	0.000	0.156	− 0.852	0.182	− 0.142
rs10918274	chr1	165714416	Intronic	T	C	TMCO1	0.99	0.976	− 0.046	1.000	0.175	0.990	0.000
rs11217878	chr11	120340383	Intronic	G	A	ARHGEF12	0.24	0.224	0.000	0.339	1.234	0.227	0.000
rs113985657	chr6	597203	Intronic	C	T	EXOC2	0.14	0.167	0.000	0.097	− 0.467	0.086	− 0.448
rs11710139	chr3	150059342	Intergenic	G	A	LINC01214;TSC22D2	0.075	0.071	0.000	0.038	− 0.586	0.045	− 0.137
rs12377624	chr9	129373110	Intergenic	G	C	MVB12B;LMX1B	0.13	0.114	0.000	0.140	0.048	0.131	0.000
rs1254276	chr14	60847001	Intergenic	C	T	LINC02322;C14orf39	0.78	0.833	0.126	0.887	1.936	0.778	0.000
rs12699251	chr7	11679113	Intronic	A	G	THSD7A	0.2	0.224	0.000	0.215	0.061	0.167	− 0.079
rs1579050	chr2	153364527	Intronic	A	G	FMNL2	0.026	0.010	0.000	0.054	0.658	0.030	0.034
rs17752199	chr6	51406848	Intergenic	A	G	TFAP2B;PKHD1	0.12	0.105	0.000	0.145	0.280	0.106	− 0.054
rs1874458	chr16	65080739	Intronic	G	A	CDH11	0.21	0.229	0.000	0.177	− 0.186	0.182	− 0.079
rs2022945	chr8	108251139	Intergenic	A	G	ABRA;ANGPT1	0.77	0.762	0.000	0.828	0.586	0.813	0.096
rs2024211	chr7	116153025	Intergenic	A	C	CAV2;CAV1	0.0089	0.005	0.000	0.011	0.061	0.015	0.079
rs2073006	chr6	637465	Intronic	C	T	EXOC2	0.15	0.181	0.046	0.118	− 0.447	0.081	− 2.070
rs2188836	chr7	117635382	Intergenic	C	T	CTTNBP2;LSM8	0.09	0.076	0.000	0.113	0.182	0.066	− 0.079
rs2317961	chr6	1533116	Intergenic	A	G	FOXF2;FOXCUT	0.51	0.519	0.000	0.500	− 0.023	0.495	− 0.009
rs2472493	chr9	107695848	Intergenic	G	A	ABCA1;SLC44A1	0.49	0.471	0.000	0.543	0.945	0.455	− 0.170
rs2472496	chr9	107695353	Intergenic	G	A	ABCA1;SLC44A1	0.47	0.457	0.000	0.538	0.544	0.449	− 0.034
rs2487032	chr9	107703934	Intergenic	G	A	ABCA1;SLC44A1	0.49	0.467	0.000	0.532	0.198	0.449	− 0.079
rs2745572	chr6	1548369	Intergenic	A	G	FOXF2;FOXCUT	0.49	0.481	0.000	0.505	0.137	0.510	0.079
rs28500712	chr4	7896213	Intronic	A	G	AFAP1	0.68	0.695	0.000	0.640	− 0.198	0.657	− 0.041
rs28520091	chr4	7846240	Intronic	C	T	AFAP1	0.29	0.329	0.000	0.328	0.199	0.313	0.041
rs28795989	chr4	7891545	Intronic	A	G	AFAP1	0.18	0.181	0.000	0.156	− 0.155	0.187	0.000
rs2935057	chr6	170454915	Intergenic	A	G	LINC00574;LOC102724511	0.76	0.700	− 0.155	0.774	0.054	0.783	0.054
rs3013274	chr6	170464367	Intergenic	G	A	LINC00574;LOC102724511	0.5	0.419	− 0.286	0.565	0.497	0.556	0.142
rs31918	chr5	14820927	Intronic	C	T	ANKH	0.33	0.319	0.000	0.344	0.046	0.298	− 0.079
rs327716	chr7	80838977	Intergenic	A	G	SEMA3C;LOC105369146	0.91	0.919	0.000	0.909	0.000	0.909	0.000
rs33912345	chr14	60976537	Exonic	C	A	SIX6	0.21	0.167	− 0.046	0.113	− 1.659	0.212	0.000
rs3785176	chr16	8896931	Intronic	A	C	PMM2	0.28	0.271	0.000	0.296	0.061	0.237	− 0.079
rs4141671	chr10	60338753	Intronic	T	C	BICC1	0.48	0.443	0.000	0.441	− 0.182	0.439	− 0.079
rs4236601	chr7	116162729	Intergenic	G	A	CAV2;CAV1	0.0099	0.005	0.000	0.016	0.172	0.015	0.079
rs55892100	chr7	115810676	Intergenic	A	G	TFEC;TES	0.8	0.805	0.000	0.785	− 0.061	0.803	0.000
rs5756813	chr22	38175477	Intergenic	G	T	TRIOBP;H1F0	0.71	0.724	0.000	0.849	2.955	0.717	0.000
rs58073046	chr11	120248493	Intronic	A	G	ARHGEF12	0.17	0.167	0.000	0.215	0.447	0.167	0.000
rs61394862	chr5	14851094	Intronic	C	T	ANKH	0.32	0.319	0.000	0.323	0.000	0.298	− 0.041
rs6478746	chr9	129367398	Intergenic	G	A	MVB12B;LMX1B	0.97	0.976	0.000	0.946	− 0.945	0.944	− 0.944
rs66602224	chr8	108293718	Intronic	G	A	ANGPT1	0.14	0.167	0.000	0.075	− 0.945	0.131	0.000
rs6732795	chr2	69411517	Intronic	A	C	ANTXR1	0.85	0.819	0.000	0.823	− 0.175	0.869	0.040
rs73174345	chr3	169252883	Intronic	T	G	MECOM	0	0.000	0.000	0.000	0.000	0.000	0.000
rs746491	chr11	86406159	Intergenic	C	A	ME3;PRSS23	0.14	0.129	0.000	0.086	− 0.715	0.096	− 0.170
rs7518099	chr1	165736880	Intronic	C	T	TMCO1	0.99	0.976	− 0.046	1.000	0.175	0.990	0.000
rs7555523	chr1	165718979	Intronic	C	A	TMCO1	0.99	0.976	− 0.046	1.000	0.175	0.990	0.000
rs7924522	chr11	128380742	Intronic	C	A	ETS1	0.86	0.876	0.000	0.742	− 2.617	0.859	0.000
rs8141433	chr22	19854006	Intergenic	A	G	GNB1L;TXNRD2	0.037	0.033	0.000	0.022	− 0.175	0.020	− 0.079
rs9284802	chr3	85095766	Intronic	G	A	CADM2	0.17	0.152	0.000	0.134	− 0.215	0.172	0.000
rs945686	chr9	129378026	Intronic	G	C	LMX1B	0.99	0.995	0.000	0.989	0.000	0.980	− 0.079
rs9494457	chr6	136474794	Intronic	T	A	PDE7B	0.33	0.262	− 0.381	0.328	0.000	0.439	2.084
rs9853115	chr3	186131600	Intergenic	T	A	DGKG;LINC02052	0.73	0.752	0.000	0.753	0.169	0.753	0.079
rs9913911	chr17	10031183	Intronic	A	G	GAS7	0.5	0.519	0.000	0.522	0.175	0.414	− 2.084

SNP ID	Chr	Position	Type	Ref Allele	Alt Allele	Nearby/containing Gene	CHB EAF	CHB log_10_ *P*	JPT EAF	JPT log_10_ *P*	KOR EAF	KOR log_10_ *P*
rs1013278	chr7	117603820	Intergenic	G	C	CTTNBP2;LSM8	0.126	0.216	0.087	− 0.081	0.114	0.413
rs10281637	chr7	116151338	Intergenic	T	C	CAV2;CAV1	0.015	0.133	0.000	− 0.232	0.007	− 0.169
rs10483727	chr14	61072875	Intergenic	T	C	SIX6;SALRNA1	0.316	4.865	0.260	1.092	0.271	3.218
rs10505100	chr8	108278616	Intronic	C	A	ANGPT1	0.272	0.285	0.303	0.819	0.283	2.435
rs10918274	chr1	165714416	Intronic	T	C	TMCO1	0.995	0.059	0.995	0.047	0.993	0.229
rs11217878	chr11	120340383	Intronic	G	A	ARHGEF12	0.189	− 0.328	0.231	− 0.023	0.227	− 0.229
rs113985657	chr6	597203	Intronic	C	T	EXOC2	0.189	0.430	0.144	0.004	0.208	4.434
rs11710139	chr3	150059342	Intergenic	G	A	LINC01214;TSC22D2	0.097	0.195	0.120	0.779	0.096	0.939
rs12377624	chr9	129373110	Intergenic	G	C	MVB12B;LMX1B	0.146	0.078	0.125	− 0.004	0.079	− 4.387
rs1254276	chr14	60847001	Intergenic	C	T	LINC02322;C14orf39	0.689	− 1.099	0.745	− 0.295	0.727	− 2.493
rs12699251	chr7	11679113	Intronic	A	G	THSD7A	0.184	− 0.059	0.207	0.023	0.229	0.876
rs1579050	chr2	153364527	Intronic	A	G	FMNL2	0.029	0.038	0.010	− 0.343	0.025	− 0.017
rs17752199	chr6	51406848	Intergenic	A	G	TFAP2B;PKHD1	0.112	− 0.059	0.115	− 0.023	0.103	− 0.624
rs1874458	chr16	65080739	Intronic	G	A	CDH11	0.228	0.078	0.212	0.000	0.250	1.588
rs2022945	chr8	108251139	Intergenic	A	G	ABRA;ANGPT1	0.733	− 0.195	0.702	− 0.779	0.723	− 2.027
rs2024211	chr7	116153025	Intergenic	A	C	CAV2;CAV1	0.015	0.133	0.000	− 0.232	0.007	− 0.169
rs2073006	chr6	637465	Intronic	C	T	EXOC2	0.189	0.430	0.183	0.440	0.220	4.434
rs2188836	chr7	117635382	Intergenic	C	T	CTTNBP2;LSM8	0.107	0.133	0.091	0.000	0.095	0.098
rs2317961	chr6	1533116	Intergenic	A	G	FOXF2;FOXCUT	0.549	0.195	0.466	− 0.313	0.475	− 0.904
rs2472493	chr9	107695848	Intergenic	G	A	ABCA1;SLC44A1	0.519	0.285	0.447	− 0.685	0.460	− 0.711
rs2472496	chr9	107695353	Intergenic	G	A	ABCA1;SLC44A1	0.485	0.059	0.438	− 0.203	0.428	− 1.306
rs2487032	chr9	107703934	Intergenic	G	A	ABCA1;SLC44A1	0.510	0.059	0.505	0.047	0.476	− 0.209
rs2745572	chr6	1548369	Intergenic	A	G	FOXF2;FOXCUT	0.451	− 0.430	0.519	0.346	0.517	0.624
rs28500712	chr4	7896213	Intronic	A	G	AFAP1	0.752	0.489	0.649	− 0.191	0.709	0.767
rs28520091	chr4	7846240	Intronic	C	T	AFAP1	0.214	− 0.598	0.279	− 0.025	0.295	0.076
rs28795989	chr4	7891545	Intronic	A	G	AFAP1	0.184	0.035	0.188	0.035	0.170	− 0.196
rs2935057	chr6	170454915	Intergenic	A	G	LINC00574;LOC102724511	0.733	− 0.133	0.793	0.236	0.729	− 0.913
rs3013274	chr6	170464367	Intergenic	G	A	LINC00574;LOC102724511	0.379	− 1.458	0.587	0.867	0.428	− 3.235
rs31918	chr5	14820927	Intronic	C	T	ANKH	0.316	− 0.051	0.375	0.339	0.334	0.054
rs327716	chr7	80838977	Intergenic	A	G	SEMA3C;LOC105369146	0.942	0.285	0.904	− 0.025	0.947	3.495
rs33912345	chr14	60976537	Exonic	C	A	SIX6	0.311	1.376	0.255	0.351	0.268	2.976
rs3785176	chr16	8896931	Intronic	A	C	PMM2	0.267	− 0.052	0.322	0.329	0.316	1.128
rs4141671	chr10	60338753	Intronic	T	C	BICC1	0.519	0.195	0.529	0.339	0.489	0.113
rs4236601	chr7	116162729	Intergenic	G	A	CAV2;CAV1	0.015	0.133	0.000	− 0.779	0.007	− 0.229
rs55892100	chr7	115810676	Intergenic	A	G	TFEC;TES	0.772	− 0.133	0.841	0.346	0.835	1.552
rs5756813	chr22	38175477	Intergenic	G	T	TRIOBP;H1F0	0.728	0.059	0.529	− 4.121	0.643	− 3.218
rs58073046	chr11	120248493	Intronic	A	G	ARHGEF12	0.155	− 0.059	0.159	− 0.035	0.168	− 0.023
rs61394862	chr5	14851094	Intronic	C	T	ANKH	0.301	− 0.059	0.365	0.339	0.333	0.196
rs6478746	chr9	129367398	Intergenic	G	A	MVB12B;LMX1B	0.971	0.000	0.990	1.200	0.967	− 0.106
rs66602224	chr8	108293718	Intronic	G	A	ANGPT1	0.189	0.430	0.144	0.004	0.204	4.208
rs6732795	chr2	69411517	Intronic	A	C	ANTXR1	0.825	− 0.133	0.894	0.489	0.900	3.633
rs73174345	chr3	169252883	Intronic	T	G	MECOM	0.000	0.000	0.000	0.000	0.000	0.000
rs746491	chr11	86406159	Intergenic	C	A	ME3;PRSS23	0.180	0.285	0.221	1.329	0.193	3.194
rs7518099	chr1	165736880	Intronic	C	T	TMCO1	0.995	0.059	0.995	0.047	0.993	0.229
rs7555523	chr1	165718979	Intronic	C	A	TMCO1	0.995	0.059	0.995	0.047	0.993	0.229
rs7924522	chr11	128380742	Intronic	C	A	ETS1	0.859	0.000	0.933	1.329	0.907	3.495
rs8141433	chr22	19854006	Intergenic	A	G	GNB1L;TXNRD2	0.029	− 0.059	0.077	1.092	0.040	0.113
rs9284802	chr3	85095766	Intronic	G	A	CADM2	0.150	− 0.099	0.255	1.296	0.176	0.106
rs945686	chr9	129378026	Intronic	G	C	LMX1B	0.990	0.000	1.000	0.339	0.987	− 0.169
rs9494457	chr6	136474794	Intronic	T	A	PDE7B	0.340	0.051	0.284	− 0.530	0.282	− 1.983
rs9853115	chr3	186131600	Intergenic	T	A	DGKG;LINC02052	0.757	0.195	0.654	− 1.329	0.701	− 0.768
rs9913911	chr17	10031183	Intronic	A	G	GAS7	0.466	− 0.285	0.572	1.376	0.554	2.055

Chr: chromosome, EAF: effect allele frequency, ref allele: reference allele, alt allele: alterative allele, CHS: Southern Han Chinese, China, CDX: Chinese Dai in Xishuangbanna, China, KHV: Kinh in Ho Chi Minh City, Vietnam, CHB: Han Chinese in Beijing, China, JPT: Japanese in Tokyo in 1000 genome project, KOR: Korean Reference Genome data base, *P*-value: adjusted Fischer’s test, statistical significance was set at *P* < 0.05 and |log_10_ *P*|> 1.301

### Composite genetic risk scores calculated using SNPs related to OAG as whole and OAG with high IOP

**Fig. 4:**
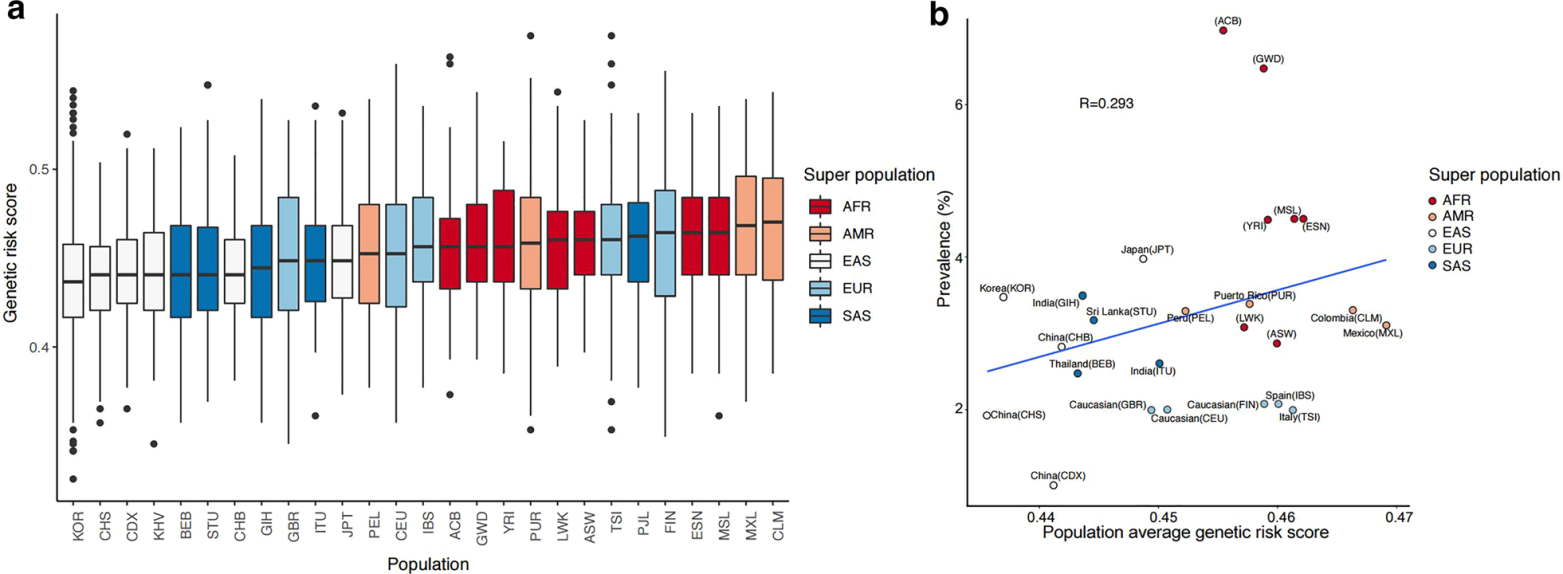
Correlation plots of the prevalence of open-angle glaucoma and genetic risk score using related single nucleotide polymorphisms. **a** The genetic risk score of OAG according to populations of different ancestry was highest in Africans, followed by Europeans, South Asians, and East Asians. **b** The prevalence of OAG was correlated with the population average genetic risk score (R = 0.293). AMR: American, EUR: Europeans, SAS: South Asians, AFR: Africans, EAS: East Asians. ACB: African Caribbean in Barbados, ASW: African Ancestry in Southwest US, BEB: Bengali in Bangladesh, CDX: Chinese Dai in Xishuangbanna, CEU: Utah residents with Northern and Western European ancestry, CHB: Han Chinese in Beijing, China, CHS: Southern Han Chinese, China, CLM: Colombian in Medellin, Colombia, ESN: Esan in Nigeria, FIN: Finnish in Finland, GBR: British in England and Scotland, GIH: Gujarati Indian in Houston, TX, GWD: Gambian in Western Division, The Gambia, IBS: Iberian populations in Spain, ITU: Indian Telugu in the UK, JPT: Japanese in Tokyo, Japan, KOR: Korean in Republic Korea, KHV: Kinh in Ho Chi Minh City, Vietnam, LWK: Luhya in Webuye, Kenya, MSL: Mende in Sierra Leone, MXL: Mexican Ancestry in Los Angeles, California, PEL: Peruvian in Lima, Peru, PJL: Punjabi in Lahore, Pakistan, PUR: Puerto Rican in Puerto Rico, STU: Sri Lankan Tamil in the UK, TSI: Toscani in Italy, YRI: Yoruba in Ibadan, Nigeria

**Fig. 5:**
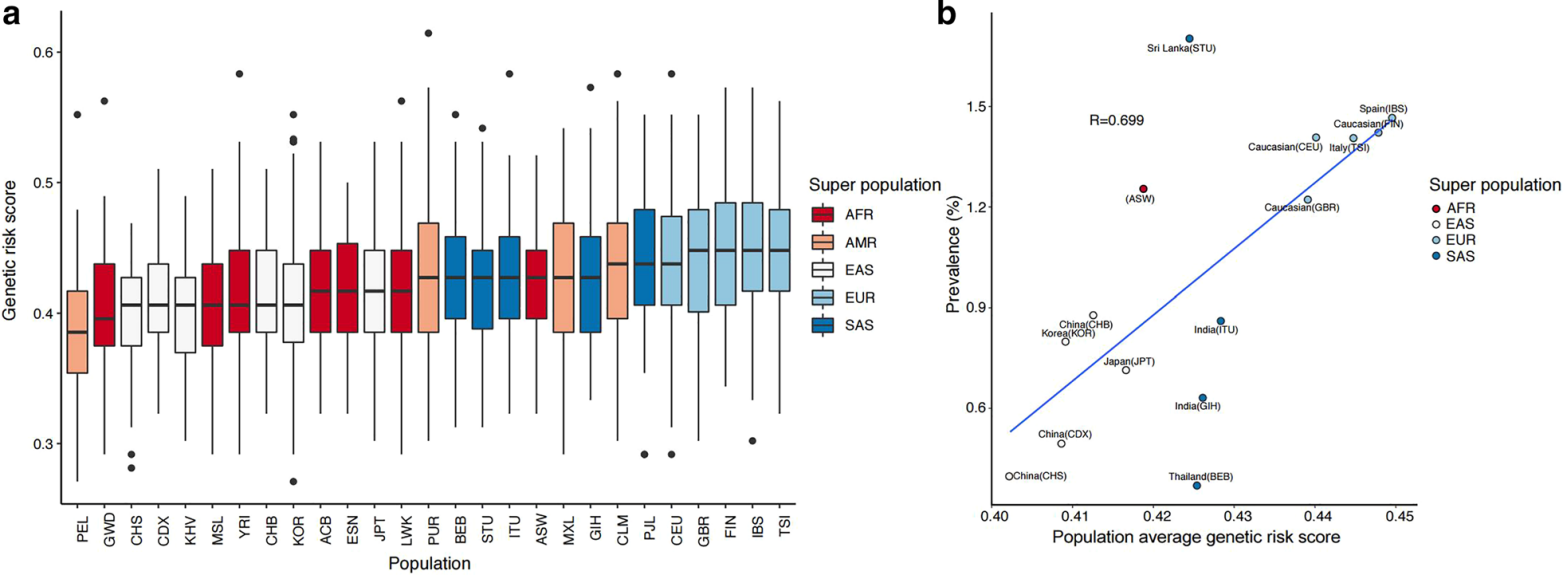
Correlation plots of the prevalence of open-angle glaucoma with intraocular pressure and genetic risk score using related single nucleotide polymorphisms. **a** The genetic risk score of OAG with high IOP according to populations of different ancestry was highest in Europeans, followed by South Asians, Africans, and East Asians. **b** The prevalence of OAG with elevated IOP was positively correlated with the population average genetic risk score (R = 0.699). The prevalence of open-angle glaucoma with normal intraocular pressure (normal-tension glaucoma) in AMR was excluded from the analysis since there were not enough research results. AMR: American, EUR: Europeans, SAS: South Asians, AFR: Africans, EAS: East Asians. ACB: African Caribbean in Barbados, ASW: African Ancestry in Southwest US, BEB: Bengali in Bangladesh, CDX: Chinese Dai in Xishuangbanna, CEU: Utah residents with Northern and Western European ancestry, CHB: Han Chinese in Beijing, China, CHS: Southern Han Chinese, China, CLM: Colombian in Medellin, Colombia, ESN: Esan in Nigeria, FIN: Finnish in Finland, GBR: British in England and Scotland, GIH: Gujarati Indian in Houston, TX, GWD: Gambian in Western Division, The Gambia, IBS: Iberian populations in Spain, ITU: Indian Telugu in the UK, JPT: Japanese in Tokyo, Japan, KOR: Korean in Republic Korea, KHV: Kinh in Ho Chi Minh City, Vietnam, LWK: Luhya in Webuye, Kenya, MSL: Mende in Sierra Leone, MXL: Mexican Ancestry in Los Angeles, California, PEL: Peruvian in Lima, Peru, PJL: Punjabi in Lahore, Pakistan, PUR: Puerto Rican in Puerto Rico, STU: Sri Lankan Tamil in the UK, TSI: Toscani in Italy, YRI: Yoruba in Ibadan, Nigeria

We calculated the composite genetic risk scores based on copies of effect alleles at OAG-associated SNPs, with the assumption that allelic associations from a significant majority of GWAS-identified variants can be replicated in non-European populations [36]. The genetic risk score of OAG was highest in Africans, followed by Europeans, South Asians, and East Asians (Fig. 4). The prevalence of OAG was correlated with the population average genetic risk score (R = 0.293, Fig. 4). In addition, the genetic risk score of OAG with high IOP was highest in Europeans, followed by South Asians, Africans, and East Asians (Fig. 5). The prevalence of OAG with elevated IOP was positively correlated with the population average genetic risk score (R = 0.699, Fig. 5).

## Discussion

Genetic factors are likely to play an essential role in the development of OAG [37]. Well-designed glaucoma cohort studies [23–26] had demonstrated the relationships of genetic loci with OAG or IOP elevation in OAG [25, 32, 33]. Although there is a Genome Aggregation Database (gnomAD) with more numbers, the authors thought that 1000 Genomes Projects would fit the design of this study, which being representative genome data and well-designed public data of whole-genome sequencing data from various populations around the world. Also, data of 1000 Genomes Projects was used for the gene target prediction model in the previous large POAG/IOP study [25]. Combining the results of these studies and the Korean whole-genome data, our study had identified the differences in allele frequencies of SNPs related to OAG or IOP elevation in OAG for worldwide populations as well as the relationship between the composite genetic risk scores and the prevalence in OAG or OAG with high IOP according to populations of diverse ancestry. The present study highlighted that the genetic risk of OAG with high IOP was present in Europeans, Americans, South Asians, Africans, and East Asians, in that order and showed a positive correlation with actual prevalence.

Our findings demonstrated that the prevalence of OAG differed among populations of diverse ancestry and was positively correlated with genetic factors. These results are consistent with those of the previous study by Kapetanakis et al*.*, which demonstrated the global variations and time trends in the prevalence of OAG [14]. In their study, among people over 80 years of age, Latinos (AMR) had the highest OAG prevalence, followed by blacks (AFR), whites (EUR), South Asians, and East Asians, and this distribution was consistent with our genetic risk score results. For people with an age of 40 years, the population prevalence of OAG was highest in Africans (4.5%), followed by Americans (3.5%), Europeans (2.1%), South Asians (2.0%), and East Asians (1.8%).

The advantage of our study is that we used the data of IOP-related SNPs in OAG, derived from a study using GERA cohort [32], a large multi-ethnic study for identifying novel loci related to IOP [33], and meta-analysis results of IGGC, ANZRAG, and UKBB [23–26]. Although NTG is considered as a type of OAG with an IOP ≤ 21 mmHg, controversy exists regarding whether NTG should be regarded as a disease within the spectrum of OAG or a distinctive disease. The focus of this study was not on the pathogenesis of NTG but on the SNPs known to be related to IOP in OAG and the prevalence of OAG with high IOP. In countries other than Asia, there are limited data on NTG proportion [12], but our study showed that the IOP-dependent mechanism in OAG could be explained according to populations of diverse ancestry. On the aspect of allele frequency, the IOP-dependent mechanism was highest in Europeans, followed by South Asians, Americans, Africans, and finally, East Asians. These results suggested that the IOP-dependent mechanism was less prominent in Koreans and Japanese.

Our results suggested that IOP-related SNPs for African ancestry might have been less enrolled, as SNPs were different because most of SNPs were derived from Europeans studies. A recent study on the association of genetic variants with OAG with individuals with African ancestry reported that rs59892895T > C risk allele was the appreciable frequency in Africans, but not in Europeans or Asians [38]. As these alleles were not included in our study, then other results would be expected, considering the prevalence of OAG in African ancestry. In addition, further studies on glaucoma cohorts of Asians and Americans are necessary to identify the differences in genetic variants with respect to those associated with African ancestry.

A recent study on multi-trait analysis of glaucoma used polygenic prediction for glaucoma progression in early manifest glaucoma cases and surgical intervention in advanced glaucoma cases, which could facilitate the development of a personalized approach for treatment [39]. In addition, another study showed that the association of polygenic risk score with IOP improved the prediction of OAG [40]. Our findings also showed a trend in genetic risk and glaucoma prevalence. These results demonstrated the importance of IOP as well as genetic factors in glaucoma development and progression. The particular contributions of the genes to the pathogenesis of OAG, however, remain to be elucidated. Moreover, the major heritable component of OAG is still unexplained. Further studies are necessary for glaucoma specialists to discover genetic variants to explain the identified associations and investigate any gene–gene or gene-environment interactions.

This study may insight the prevalence difference of OAG and NTG according to region and country. In European descent, most glaucoma is known to be POAG with high IOP, and POAG with normal IOP is known to be the minority. However, in East Asians, especially in Japan and Korea, most of the OAG is NTG. Since glaucoma treatment is a management of lowering IOP and other risk factors, understanding the genetic difference in SNP frequencies with IOP would provide sufficient insights regarding the pathogenesis of glaucoma for clinical ophthalmologists and glaucoma specialists. A major strength of our study was the inclusion of the large Korean whole-genome data (n = 1722) to reflect the allele frequency of SNPs related to OAG and OAG with high IOP. Additionally, we did not systematically organize the new glaucoma cohort and analyze the effects; instead, we compared the 1000 Genomes Project data with OAG-related SNP data from the GWAS catalog. However, there are a few limitations to this study. First, the GWAS catalog contains data for which the risk allele is not clearly defined in the minor allele frequency (MAF). However, we did not exclude these in our study because the majority of MAFs are likely to be risk alleles, so removing all of the undefined alleles would result in inaccurate subgroup analysis. Further study is needed for the data curation of 32 undefined SNPs. To solve this problem, risk allele curation is necessary for the GWAS catalog, based on further results of large population studies using glaucoma cohorts. Second, the statistical significance of EAF in Koreans was high and should be interpreted with caution since the Fisher’s test can decrease the *P*-value as the number of subjects increases, even with the same odds ratio values as the Korean reference genome number was 1722, which was very high, assuming that the genome number of 26 populations belonging to the 1000 Genomes Project was about 100, ranging from 61 to 113. Third, we used the composite risk score that did not include the effect size weights, as the weighted-odd ratios vary according to the ethnic group even for the same SNP, and there are inaccuracies due to insufficient study data on OAG-related SNPs in the Africans and Asians population. In the future, the polygenic risk score with the effect size weighted odd ratio will be a more desirable study. Fourth, our study analyzed what is currently known; it is not a study that shows new genetic loci or pathway analysis using cell culture and animal study. Additionally, our findings should be interpreted in consideration of the fact that the penetrance is variable even when causative SNPs are present in specific individuals. Nevertheless, it would be meaningful to understand genetic research from the point of view of a clinical glaucoma specialist.

## Conclusions

Our study showed substantial population differentiation in allele frequencies in both of OAG-related SNPs and IOP-related SNPs in OAG. From the allele frequency of these SNPs, we calculated the composite risk scores for OAG and OAG with high IOP for 26 ethnic groups in the 1000 Genomes Project and Koreans. In addition, the prevalence of OAG and OAG with high IOP correlated with genetic risk scores. We observed differences in allele frequencies associated with SNPs related to IOP in OAG between Koreans and other populations of diverse ancestry, which may explain the high prevalence of OAG with normal IOP predominantly in Koreans and East Asians.

## Supplementary Information

**Additional file 1.** **Figure S1.** A heatmap generated using open-angle glaucoma-related single nucleotide polymorphisms in the global population and the East Asian population. **Figure S2.** A Heatmap generated using open-angle glaucoma-related single nucleotide polymorphisms in the East Asian population

**Additional file 2.** **Table S1.** Effect allele frequencies (EAFs) of open-angle glaucoma related single nucleotide polymorphisms in populations of diverse ancestry including Koreans.

**Additional file 3.** **Table S2.** Effect allele frequencies (EAFs) of open-angle glaucoma-related single nucleotide polymorphisms in East Asian groups, including Koreans.

## Data Availability

The raw datasets generated and analyzed during the current study are not publicly available since any data providing the whole-genome sequencing data is considered to be personal property by the Korea Bioethics law. However, the raw whole-genome sequencing data for research are available at the reasonable request under the permission of the National Biobank of Korea contact at [http://nih.go.kr/biobank/cmm/main/mainPage.do?/] and e-mail [biobank@korea.kr]. The allele frequency of Korea reference genome data base (KRGDB) is available [http://152.99.75.168:9090/KRGDBDN/dnKRGinput.jsp], files required are all three of ‘the totally merged sets’ of common variants, rare variants, and indels. The 1000genomes data is available, all the files from the following folder were downloaded, [ftp://ftp.1000genomes.ebi.ac.uk/vol1/ftp/release/20130502/] (last accessed: January 15, 2020). The genome-wide association study (GWAS) catalog data is available in the (NHGRI-EBI, [https://www.ebi.ac.uk/gwas/docs/file-downloads], “All associations v1.0.2—with added ontology annotations, GWAS Catalog study accession numbers and genotyping technology”, December 2019).

## Abbreviations

AFR: African
AMR: American
ANZRAG: Australian and New Zealand Registry of Advanced Glaucoma
EAFs: Effect allele frequencies
EAS: East Asian
EUR: European
GWAS: Genome-wide association studies
IGGC: International Glaucoma Genetics Consortium
IOP: Intraocular pressure
ISGEO: International Society of Geographical and Epidemiological Ophthalmology
KOR: Korean
KRGDB: Korean Reference Genome Database
MAF: Minor allele frequency
NTG: Normal-tension glaucoma
OAG: Open-angle glaucoma
SAS: South Asian
SNP: Single nucleotide polymorphism
UKBB: UK Biobank
