# Supplementary material for: Analysis of risk allele frequencies of single nucleotide polymorphisms related to open-angle glaucoma in different ethnic groups
Source: BMC Med Genomics. 2021 Mar 16;14:80. doi: 10.1186/s12920-021-00921-2 (PMC7962394; doi:10.1186/s12920-021-00921-2)
Supplement: Supplementary file 1 — Additional file 1. Figure S1. A heatmap generated using open-angle glaucoma-related single nucleotide polymorphisms in the global population and the East Asian population. Figure S2. A Heatmap generated using open-angle glaucoma-related single nucleotide polymorphisms in the East Asian population [file 12920_2021_921_MOESM1_ESM.docx]

**Fig S1.** A heatmap generated using open-angle glaucoma-related single nucleotide polymorphisms in the global population

**
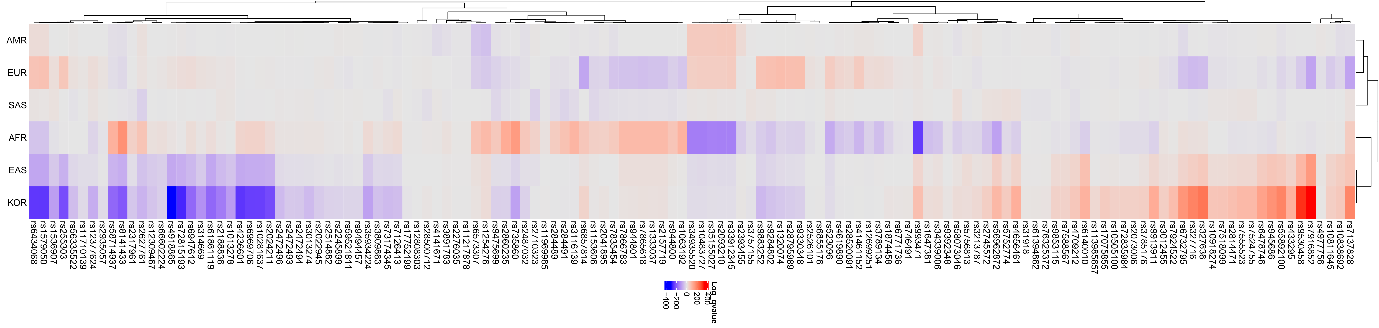
**

The heatmap shows how significantly the effect alleles are enriched or depleted in each population. Each row shows SNPs, and each column shows populations of diverse ancestry. Red color means effect allele is enriched, whereas purple color means effect allele is depleted (log_10_ *P* > 1.301 indicated enrichment, log_10_ *P* < − 1.301 indicated depletion). A hierarchical clustering tree shows the differences among continents; EUR, AMR, and SAS are in one cluster, and AFR, EAS, and KOR are in another cluster.

AMR: American, EUR: Europeans, SAS: South Asians, AFR: Africans, EAS: East Asians, KOR: Koreans

**Fig S2.** A Heatmap generated using open-angle glaucoma-related single nucleotide polymorphisms in the East Asian population


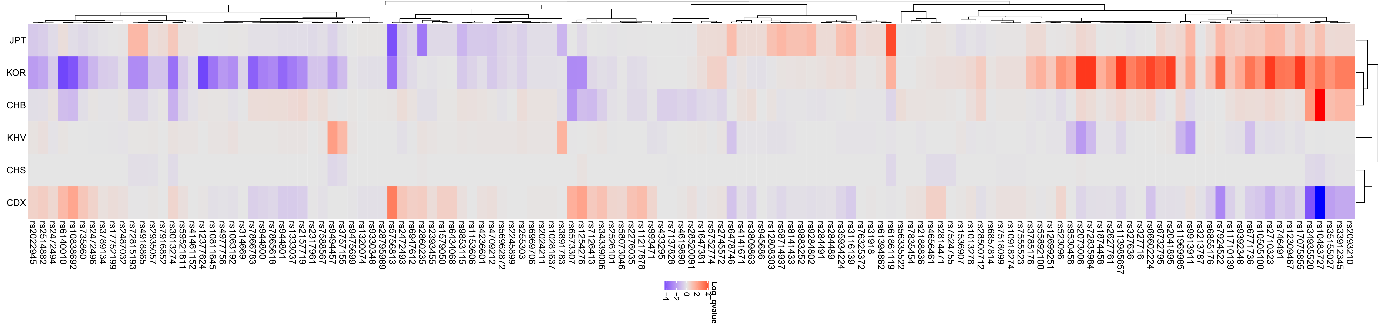


The heatmap shows how significantly the effect alleles are enriched or depleted in each population. Each row shows SNPs, and each column shows populations of diverse ancestry. Red color means effect allele is enriched, whereas purple color means effect allele is depleted (log_10_ *P* > 1.301 indicated enrichment, log_10_ *P* < − 1.301 indicated depletion). A hierarchical clustering tree shows the differences among East Asians, KOR, JPT, and CHB in one cluster, and CHS, and CDX in another cluster.

CDX: Chinese Dai in Xishuangbanna, CHB: Han Chinese in Beijing, China, CHS: Southern Han Chinese, China, JPT: Japanese in Tokyo, Japan, KOR: Korean in Republic Korea, KHV: Kinh in Ho Chi Minh City, Vietnam
